# A Review of Orthopaedic Surgical Set-Up and Introduction of the TULIPS Mnemonic – Six Simple Steps for Optimising Set-Up in Orthopaedic Surgery

**DOI:** 10.7759/cureus.9806

**Published:** 2020-08-17

**Authors:** Gregory Neal-Smith, David S Kim, Alexander Wood

**Affiliations:** 1 Trauma and Orthopaedics, Oxford University Hospitals NHS Foundation Trust, Oxford, GBR; 2 Trauma and Orthopaedics, Oxford University Medical School, Oxford, GBR; 3 Trauma, Oxford University Hospitals NHS Foundation Trust, Oxford, GBR

**Keywords:** orthopaedics, surgery, set-up, operating theatre, mnemonic, quality improvement

## Abstract

Conducting a thorough check to ensure that all equipment and personnel are positioned correctly at the start of any operation is essential for both the safety of the surgical team and the patient outcome. Orthopaedic surgery in particular carries a high risk of occupational injury and this group could benefit greatly from ergonomic improvements. This review highlights multiple factors that can influence safety of surgeons, surgical efficiency and patient outcomes.

“TULIPS” is a mnemonic that lists six key steps in optimising the surgical procedure through effective positioning of equipment and personnel pre-operatively. This was trialled by distribution amongst orthopaedic registrars regionally and it received excellent feedback, with the majority changing their current practice. Here we report that using this simple and memorable checklist can assist orthopaedic surgeons in setting up the operating theatre, facilitating ergonomic improvements that can reduce the risk of musculoskeletal injury and radiation exposure.

## Introduction

Set-up for surgical procedures is essential for the safety of the patient, the surgeon and patient outcomes. It has been suggested orthopaedic surgeons would benefit most from ergonomic improvements, as they are particularly at risk of occupational injury [1]. Data by Davis, et al. has shown that a high proportion of orthopaedic surgeons sustain one or more injuries at the workplace during their career [1]. Efforts have been made to improve the ergonomic experience and reduce the strain that occurs during surgery, but adherence to these techniques has not been widely adopted. This has been attributed to general lack of awareness of the current recommendations within the surgical specialties [2]. It has been recommended to raise awareness of these injuries and also devote institutional resources to help combat these issues in the orthopaedic community [2].

The checklist method has been a proven approach across both medical and non-medical disciplines including aviation, from where many of our current protocols in healthcare were developed from [3]. Implementing checklists in the surgical setting has enabled significant improvements in patient in-hospital mortality, exemplified by the World Health Organization (WHO) surgical checklist [4]. Here we report the successful implementation of our own checklist approach, TULIPS, as a method of improving the surgical set-up prior to operations.

## Materials and methods

A survey was sent to all the Trauma and Orthopaedic (T&O) registrars working in the Oxfordshire deanery before the introduction of the TULIPS mnemonic. The aim of the survey was to assess whether surgical teams had a methodological approach to setting up the operating theatre, and whether individuals had experienced problems in setting up in theatre.

Participants were then asked to trial the TULIPS mnemonic in their daily practice as a checklist for setting up the operating theatre prior to their surgical procedures over a period of two weeks. After this period, participants were contacted with a second survey for feedback on its impact in practice.

TULIPS mnemonic

T - Table and Tourniquet position U - You and Your position L - Lighting position I - Imaging and Image Intensifier position P - Patient and Personnel position S - Screen Image position

## Results

A total of 14 responses were received in our pre-intervention survey, and 14 responses (all initial participants) in our post intervention survey.

Pre-intervention survey results

8/14 (57%) registrars reported they had a method of setting up the operating theatre, however only 2/14 (14%) reported using a checklist approach to help them set-up. 12/14 (86%) reported having experienced difficulties in surgery directly linked to poor theatre set-up, with 8/14 (57%) all mentioning that image intensifier position had been problematic. Other frequent comments related to all other areas of the TULIPS mnemonic, including table position, their position, staff positioning and lighting. Of the 12 who had difficulties due to their surgical set-up, 9/12 (75%), did not have a formal checklist approach. Of the eight surgeons who had a method for setting-up, only 3/8 (38%) used a formal checklist approach, and 6/8 (75%) still had difficulties with their surgical set-up.

Post-intervention survey results

After a trial phase of two weeks, 13/14 (93%) reported finding TULIPS useful. This included all 12/14 (86%) from the pre-intervention survey who had previously reported experiencing difficulties in surgery. 13/14 (93%) reported that it had made them reconsider their surgical set-up. 8/14 (57%) stated TULIPS had changed their practice for setting-up in theatre and they now incorporated this checklist approach into their standard practice.

6/14 (43%) did not change their practice as a result of TULIPS, however the pre-intervention survey showed 5/6 (83%) of those that did not change their practice already had a method of surgical set-up prior to the two week trial of TULIPS.

Out of the 8/14 (57%) who reported having a current method for setting up in theatre, all 8/8 (100%) reported TULIPS to be a useful method of approaching set-up, of which 7/8 (88%) also reported that TULIPS made them reconsider their own set-up, with 3/8 (38%) still choosing to change their practice permanently as a result.

Most importantly, of the 6/14 (43%) who reported in the pre-intervention survey that they did not have a method of surgical set-up, 5/6 (83%) changed their practice permanently because of TULIPS.

## Discussion

TULIPS is a simple, memorable mnemonic that can be adopted in operating theatres and is easily understood and implemented by surgeons. Our results show that the TULIPS method of approaching surgical set-up was effective in changing practice and serves as a useful checklist for orthopaedic surgeons. The introduction of TULIPS regionally has been adopted into standard practice prior to operations for those who do not already have a similar method in place. 

Table and tourniquet position

Correct positioning of the operating table has been shown to benefit the surgeon by providing proper ergonomic support and reducing the risk of occupational musculoskeletal injuries common across all surgical disciplines [5]. Adjusting the height of the table prior to surgery can reduce physical fatigue for the individuals involved and therefore create a safer working environment for them and the patient [6]. Secondly, the choice of table and its associated advantages and disadvantages need to be considered for every patient. Traction tables are commonly used in lower limb trauma and these allow indirect fracture reduction and maintenance of reduction. They do however have associated risks such as perineal integument injuries, limb malalignment and pudendal nerve injury [7]. Alternative considerations could include a radiolucent table with manual traction being applied, or periodic release of traction to help avoid these complications [7].

Tourniquets are commonly used in limb trauma surgery to exsanguinate blood flow to a distal extremity. The decision to use tourniquets, along with its location and timing also has important effects on patient outcome [8]. A systematic review by Præstegaard, et al. found that length of hospital stay was longer in groups who had tourniquet use compared to non-tourniquet groups in lower limb fracture surgery [8]. The pressure generated in modern tourniquets, particularly if hyperinflated, can lead to a number of complications including nerve trauma and rhabdomyolysis [9,10]. Under-pressurised cuffs cause insufficient exsanguination and can result in excess intraoperative bleeding. The cuff should also not overlie bony prominences, such as the head of the fibula, in order to avoid nerve compression injury. Further considerations should be made in patients with relative complications such as severe infections, poor cardiac reserve, Raynaud’s disease, or peripheral neuropathy [11]. It is therefore critical to consider the use, along with placement and timing of the tourniquet prior to the procedure.

You and your position

Correct positioning of the surgeon facilitates better economy of movement, visualisation of anatomical structures and comfort. In one study, surgeons were found to be rotating more than 50% from their midline or stretching to 50% of their limit, for a third of the duration of the operation [12]. Ideally, the surgeon’s arms should be positioned such that their elbows rest at their sides with forearms parallel to the floor in the neutral position, for comfort and to enable a maximum range of vertical movement with the instruments [12]. Standing in close proximity to the surgical site should enable both better control and when suturing, lesser requirement for rotating instruments through larger angles to achieve the same suture [13]. Surgeon discomfort may contribute to surgical errors and therefore, ergonomic considerations are likely beneficial to both the surgeon and the patient. Also, failure to communicate or cooperate with others in the surgical team, has been associated with a higher incidence of adverse events [14]. Standing closer to other surgeons in the team enables clearer verbal communication during the procedure. Allocating specific roles to each person around the table according to their position with respect to the patient, facilitates effective division of labour and concurrent activity. This also minimizes the need to reach over or sustain uncomfortable positions for prolonged periods of time which increases risk of musculoskeletal strain.

Lighting position

All lighting systems in the operating room must be positioned to adequately illuminate the surgical area, prevent glare and provide true colour rendering [15]. These are critically important in helping determine key anatomical structures as well as the condition of the patient. Poor lighting in the operating room has been associated with both risks to patient safety and an avoidable occupational hazard to theatre staff [15]. Furthermore, it is important that the light position achieves this without compromising sterility or obstructing the movement of the surgeon or their assistant. Ambient light has the potential to reduce clarity on any screens used in theatre and cause glare by reflecting into the surgeon’s eyes. Light sources placed behind the screen cause pupillary constriction and therefore are ideally placed at a right angle to the line between the screen and the surgeon, or instead dimmed appropriately.

Reducing the handling of light handles is also suggested as they can carry a significant level of bacterial contamination. It is important the surgeon considers the lighting position preoperatively and pre-empts any movements needed, which could be performed by a colleague to reduce chance of contamination

Imaging and image intensifier position

Fluoroscopy is a valuable imaging tool used frequently in orthopaedic surgery. This is composed of an X-ray tube, an image intensifier, a collimator and a display. Given the associated risks from radiation exposure, it is important to encourage surgical teams during set-up to adhere to protocols that limit this as much as possible [16]. Many factors are known to reduce the level of radiation exposure including correct positioning of the c-arm and personal protective equipment (PPE) usage [16]. Tremains et al. found evidence to suggest that adopting an inverted position during intraoperative fluoroscopy reduces radiation exposure [17]. By using the image intensifier as a table and facing the x-ray tube up, the radiation exposure to the patient is reduced by 59% and to the surgeon’s head, body and groin by 67%, 45% and 15% respectively [17]. This is in comparison to a normal configuration, where the image intensifier is positioned at the top of the c-arm and the x-ray tube is down [17]. This demonstrates the importance of considering the position of the image intensifier in a manner which reduces the scattering of the x-ray beam.

Orthopaedic surgeons have been shown to have an increased risk of cancer that has partly been attributed to occupational radiation exposure from theatre c-arm fluoroscopy [18]. Reducing radiation exposure from the image intensifier by following correct positioning protocols such as the ‘As Low As Reasonably Achievable’ (ALARA) principle, is pertinent to promoting safe practice in orthopaedic surgery [19]. One study found that unprotected individuals working closer than 24 inches from the fluoroscopic beam received significant amounts of radiation, compared to those working 36 inches away or further [20].

In surgery involving regular c-arm repositioning, its placement should facilitate the adjustment for imaging the required structures, and its movement should be as unobstructed as possible. For example, insertion of dynamic hip screws in the proximal femur requires regular anteroposterior (AP) and lateral views. The c-arm is positioned obliquely between the legs of the patient to enable an axial view and must then be tilted by 10-15 degrees to compensate for the anteversion of the femoral neck [21]. Similarly, in osteosyntheses of the proximal humerus, the patient is positioned in the beach-chair position, resulting in the upper arm already tilted towards the floor. The C-arm must be tilted to compensate such that the central beam remains perpendicular to the humeral shaft in order to get the desired AP and lateral views [21].

Lastly, pre-operative imaging must also be considered at this stage on the checklist. This ensures nothing blocks the field of view, and therefore fracture patterns and angles can be clearly seen on previous x-rays to help plan the surgical approach.

Patient and personnel position

Positioning of the patient correctly on the surgical table directly impacts patient outcomes. In one study, surgical positioning led to serious but preventable injuries. Of the 172 patients, 12.2% experienced injuries resulting from their surgical positioning on the table including peripheral nerve injuries, pain around pressure points, and erythema [22]. A pressure of as little as 70mmHg exerted in an area of skin for longer than two hours has been shown to be enough to induce irreversible ischemia of the soft tissue. Susceptible areas include the forehead, iliac crests, and bony prominences [23]. It is therefore important to consider correct table positioning as a key part of patient positioning that will reduce the risk of musculoskeletal, neurological, and soft tissue injuries. Further to this, adjuncts used alongside the patient such as silicone gel positioners or radiolucent triangles may ease pressure points on the table. For example, the prone position without additional padding on the table significantly increases pressure on the thorax and abdomen. Abdominal compression raises intra-abdominal pressure which directly exerts on the inferior vena cava and causes reduced venous return [24,25]. These physiological changes arise directly as a result of the patient’s position on the table and must therefore be considered prior to the operation. This would ideally be discussed preoperatively between the surgeon and the anaesthetist as these issues are relevant to both individuals.

In a survey across nine laparoscopic surgical procedures, 85 items were recorded in the log of encountered problems, of which 57 related directly to the positioning of equipment and personnel. Both surgeons and nurses experience substantial musculoskeletal strain resulting from fixed postures for prolonged periods of time [26]. Whilst this is mostly inevitable due to the nature of the task, some of this strain could be mitigated by correctly positioning individuals to ensure that there is less straining in order to have good surgical site access, view screens, avoid glare from lighting, or access instruments. This was highlighted in a study that found surgeons and their surgical assistants had worse postures and higher ergonomic stress loads [26]. 

Secondly, effective communication between members of the team and efficient division of labour is owed to a good awareness of everyone’s positioning in the room. Additionally, all individuals present in the theatre must have a good awareness of wires and other trip hazards, as well as an appreciation for the demarcation between sterile and non-sterile areas. Correctly positioning everyone prior to the operation is important in enabling good teamwork which will reduce the risk of preventable adverse outcomes.

Screen image position

The optimal positioning for any video display terminal was shown to be between 10-25 degrees below the line of sight [27]. Neck stiffness and pain are commonly noted amongst surgeons who regularly conduct laparoscopic procedures [28]. If ergonomic set up were the sole consideration in laparoscopic surgery, the ideal positioning of the monitor for the surgeon would be directly in the surgeon’s line of sight - between the surgeon and the surgical field, with a second monitor directly at eye-level in front [29]. Surgeons have significantly lower neck strain when monitors are placed directly ahead at eye level as evidenced by lower electromyographic activity of the main neck muscles [30]. Though new devices are emerging on the market to enable screens to be placed in positions better suited for the surgeon’s comfort, most operating theatres use a standard video display with limited manoeuvrability. Therefore, the screen should be positioned such that the angle cast from the display to the surgeon to the surgical site, is minimised where possible.

## Conclusions

In conclusion, this review has highlighted multiple theatre set-up factors that can influence patient outcome, surgical efficiency, and safety of surgeons. These include table, personnel, and equipment positioning. Better theatre set-up has been shown to improve patient outcomes and surgical efficiency and reduces risk of occupational injury and physical fatigue of the surgeon.

We recommend the use of this mnemonic as a checklist during pre-operative set-up to promote surgical best practice. Incorporating a method of remembering six key tasks into routine practice may provide a simple but effective tool to avoid preventable outcomes and Never Events caused by incorrect set-up prior to the operation.

## Appendices

ACKNOWLEDGEMENT

To Timothy White, Consultant Orthopaedic surgeon at the Royal Infirmary of Edinburgh, whom first introduced AW to the TULIPS mnemonic.
